# Supplementary material for: Gestational CBD Shapes Insular Cortex in Adulthood
Source: Cells. 2024 Sep 4;13(17):1486. doi: 10.3390/cells13171486 (PMC11394289; doi:10.3390/cells13171486)
Supplement: Supplementary file 1 [file cells-13-01486-s001.zip › cells-3111700-supplementary.pdf]

Table S1. Multivariate analysis of the compound effects of gestational CBD exposure

CONDITION	Measure	Membrane Capacitance	Rheobase	Resting Potential	Input Resistance -400	Input Resistance +100	APs numeber 150 pa
aIC SHAM MALE	Membrane Capacitance	1.00	0.17	-0.18	0.24	-0.33	-0.12
	Rheobase	0.17	1.00	-0.34	0.67	-0.67	-0.71
	Resting Potential	-0.18	-0.34	1.00	-0.31	0.05	-0.02
	Input Resistance -400	0.24	0.67	-0.31	1.00	-0.85	-0.72
	Input Resistance +100	-0.33	-0.67	0.05	-0.85	1.00	1.00
	APs numeber 150 pa	-0.12	-0.71	-0.02	-0.72	0.89	1.00
		Membrane Capacitance	Rheobase	Resting Potential	Input Resistance -400	Input Resistance +100	APs numeber 150 pa
pIC SHAM MALE	Membrane Capacitance	1.00	0.66	-0.47	0.50	-0.20	-0.60
	Rheobase	0.66	1.00	-0.65	0.58	-0.15	-0.90
	Resting Potential	-0.47	-0.65	1.00	-0.13	-0.31	0.58
	Input Resistance -400	0.50	0.58	-0.13	1.00	-0.65	-0.63
	Input Resistance +100	-0.20	-0.15	-0.31	-0.65	1.00	0.26
	APs numeber 150 pa	-0.60	-0.90	0.58	-0.63	0.26	1.00
		Membrane Capacitance	Rheobase	Resting Potential	Input Resistance -400	Input Resistance +100	APs numeber 150 pa
aIC SHAM FEMALE	Membrane Capacitance	1.00	-0.36	0.17	0.05	0.16	0.33
	Rheobase	-0.36	1.00	0.26	0.49	-0.67	-0.60
	Resting Potential	0.17	0.26	1.00	-0.06	0.17	0.12
	Input Resistance -400	0.05	0.49	-0.06	1.00	-0.78	-0.29
	Input Resistance +100	0.16	-0.67	0.17	-0.78	1.00	0.55
	APs numeber 150 pa	0.33	-0.60	0.12	-0.29	0.55	1.00
		Membrane Capacitance	Rheobase	Resting Potential	Input Resistance -400	Input Resistance +100	APs numeber 150 pa
pIC SHAM FEMALE	Membrane Capacitance	1.00	0.44	0.07	0.39	-0.43	-0.51
	Rheobase	0.44	1.00	-0.36	0.77	-0.51	-0.96
	Resting Potential	0.07	-0.36	1.00	-0.13	-0.20	0.38
	Input Resistance -400	0.39	0.77	-0.13	1.00	-0.71	-0.77
	Input Resistance +100	-0.43	-0.51	-0.20	-0.71	1.00	0.50
	APs numeber 150 pa	-0.51	-0.96	0.38	-0.77	0.50	1.00
		Membrane Capacitance	Rheobase	Resting Potential	Input Resistance -400	Input Resistance +100	APs numeber 150 pa
aIC CBD MALE	Membrane Capacitance	1.00	0.25	-0.56	0.52	-0.19	-0.17
	Rheobase	0.25	1.00	-0.51	0.62	-0.96	-0.80
	Resting Potential	-0.56	-0.51	1.00	-0.71	0.56	0.40
	Input Resistance -400	0.52	0.62	-0.71	1.00	-0.54	-0.38

	Input Resistance +100	-0.19	-0.96	0.56	-0.54	1.00	0.82
	APs numeber 150 pa	-0.17	-0.80	0.40	-0.38	0.82	1.00

		Membrane Capacitance	Rheobase	Resting Potential	Input Resistance -400	Input Resistance +100	APs numeber 150 pa
pIC CBD MALE	Membrane Capacitance	1.00	-0.20	0.07	0.21	0.09	0,17
	Rheobase	-0.20	1.00	-0.55	0.25	-0.70	-0,89
	Resting Potential	0.07	-0.55	1.00	0.17	0.19	0,50
	Input Resistance -400	0.21	0.25	0.17	1.00	-0.60	-0,36
	Input Resistance +100	0.09	-0.70	0.19	-0.60	1.00	0,71
	APs numeber 150 pa	0.17	-0.89	0.50	-0.36	0.71	1.00

		Membrane Capacitance	Rheobase	Resting Potential	Input Resistance -400	Input Resistance +100	APs numeber 150 pa
aIC CBD FEMALE	Membrane Capacitance	1.00	-0.04	0.27	0.11	-0.12	-0.02
	Rheobase	-0.04	1.00	-0.90	0.54	-0.82	-0.89
	Resting Potential	0.27	-0.90	1.00	-0.55	0.64	0.72
	Input Resistance -400	0.11	0.54	-0.55	1.00	-0.65	-0.48
	Input Resistance +100	-0.12	-0.82	0.64	-0.65	1.00	0.79
	APs numeber 150 pa	-0.02	-0.89	0.72	-0.48	0.79	1.00

		Membrane Capacitance	Rheobase	Resting Potential	Input Resistance -400	Input Resistance +100	APs numeber 150 pa
pIC CBD FEMALE	Membrane Capacitance	1.00	0.18	-0.12	0.19	-0.19	-0.17
	Rheobase	0.18	1.00	-0.70	0.82	-0.82	-0.79
	Resting Potential	-0.12	-0.70	1.00	-0.58	0.62	0.75
	Input Resistance -400	0.19	0.82	-0.58	1.00	-0.87	-0.72
	Input Resistance +100	-0.19	-0.82	0.62	-0.87	1.00	0.87
	APs numeber 150 pa	-0.17	-0.79	0.75	-0.72	0.87	1.00

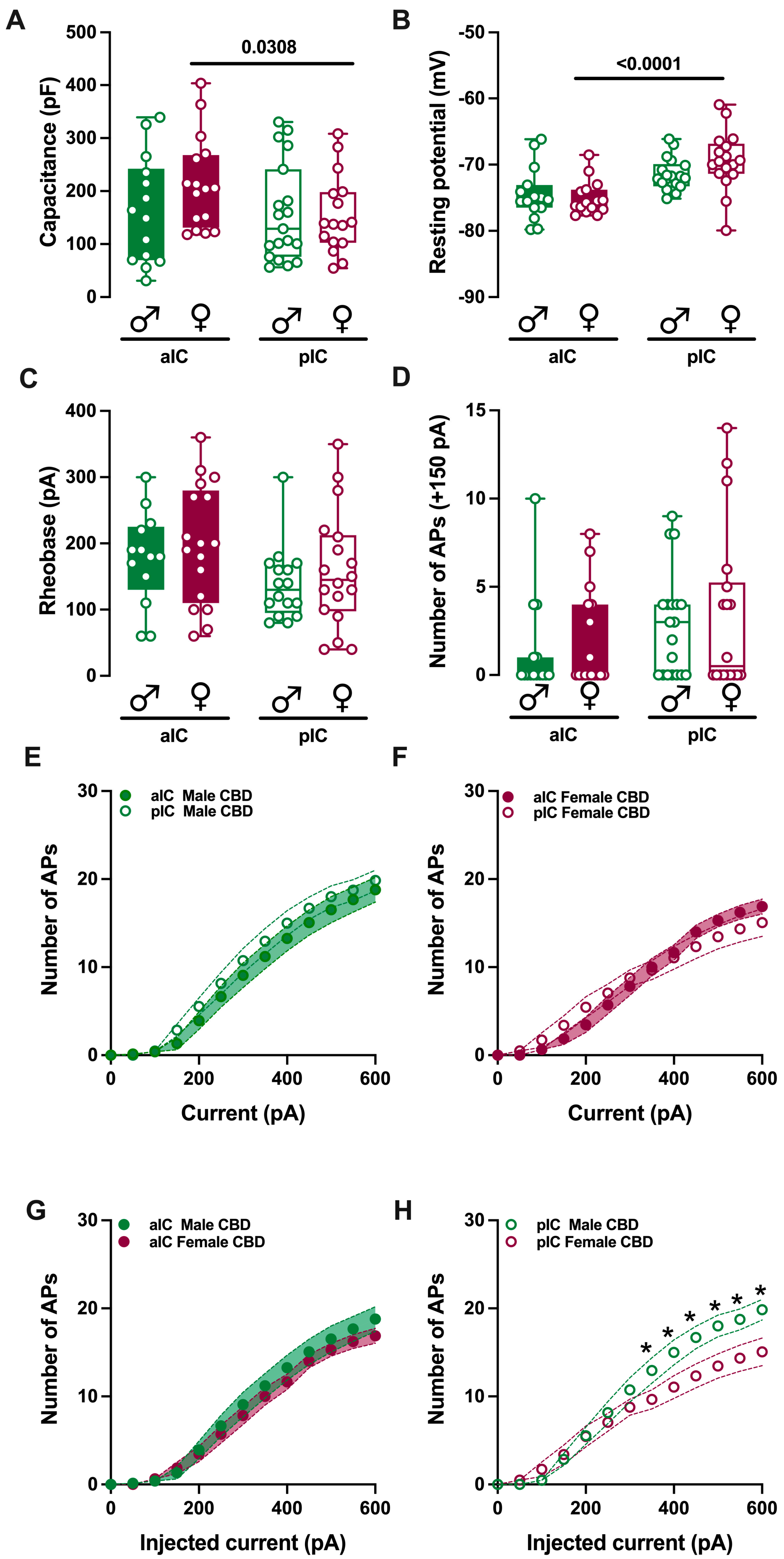

A
Capacitance (pF)
0.0308
aIC
pIC
B
Resting potential (mV)
<0.0001
C
Rheobase (pA)
D
Number of APs (+150 pA)
E
aIC Male CBD
pIC Male CBD
Number of APs
Current (pA)
F
aIC Female CBD
pIC Female CBD
G
Injected current (pA)
H

Supplemental Figure S1. Prenatally exposure to CBD induces sex and region-specific alteration in Insular Cortex of CBD-exposed mice. (A) Quantitative analysis of passive membrane properties of CBD-exposed male and females unveiled that across IC subregions, pyramidal neurons of aIC are bigger compared to those in pIC only female progeny, but not in male. (B) The resting membrane potential of aIC pyramidal neurons is more hyperpolarized than those of pIC in noly CBD-exposed female. (C) Pyramidal neurons of both IC did not differ in the rheobase as well as in the numbers of APs evoked at +150 pA (D). (E-F) The number of evoked action potentials in response to depolarizing current steps were similar across IC subregions in both sexes. (G) CBD-exposed progeny showed a comparable excitability within the aIC. (H) In contrast the pIC of CBD female showed a lower excitability compared to the pIC of CBD male. Data are presented as box-and-whisker plots (minimum, maximum, median) for (A-B-C-D) and as mean ± SEM in XY plot for (E-F-G-H). Two-way ANOVA followed by Šídák's multiple comparison test was performed for (A-B-C-D), while Mann-Whitney U test was applied for (E-F-G-H). P–values < 0.05 depicted in the graph. The sample size expressed as cells/Animal is: aIC CBD male = 15/8, pIC CBD male = 19/9, aIC CBD female = 17/7, pIC CBD female = 17/6.

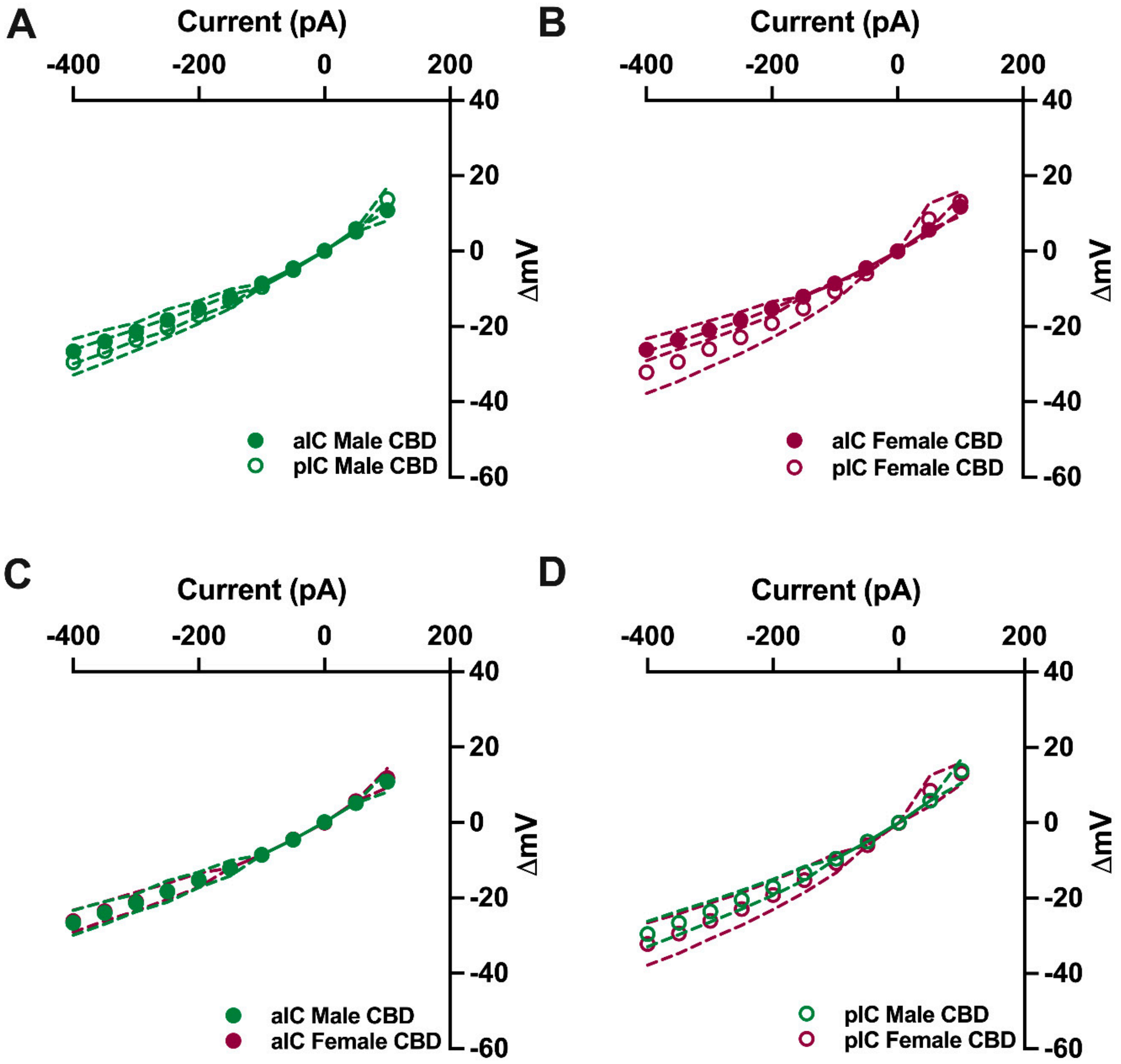

Supplemental Figure S2. Prenatal CBD exposure does not change the membrane voltage response of IC pyramidal neurons. (A-D) In response to current injections of 50 pA from -400 pA to +50 pA the membrane profile of pyramidal neurons was similar across and within IC subregions in both sexes. Data are presented as mean ± CI in XY plots for (A-D). A Mann-Whitney U test was used for the statistical analysis, and *p-value < 0.05 was considered significant. The sample size expressed as cells/Animal is: aIC CBD male = 15/8, pIC CBD male = 20/9, aIC CBD Female = 13/6, pIC CBD Female = 18/8.

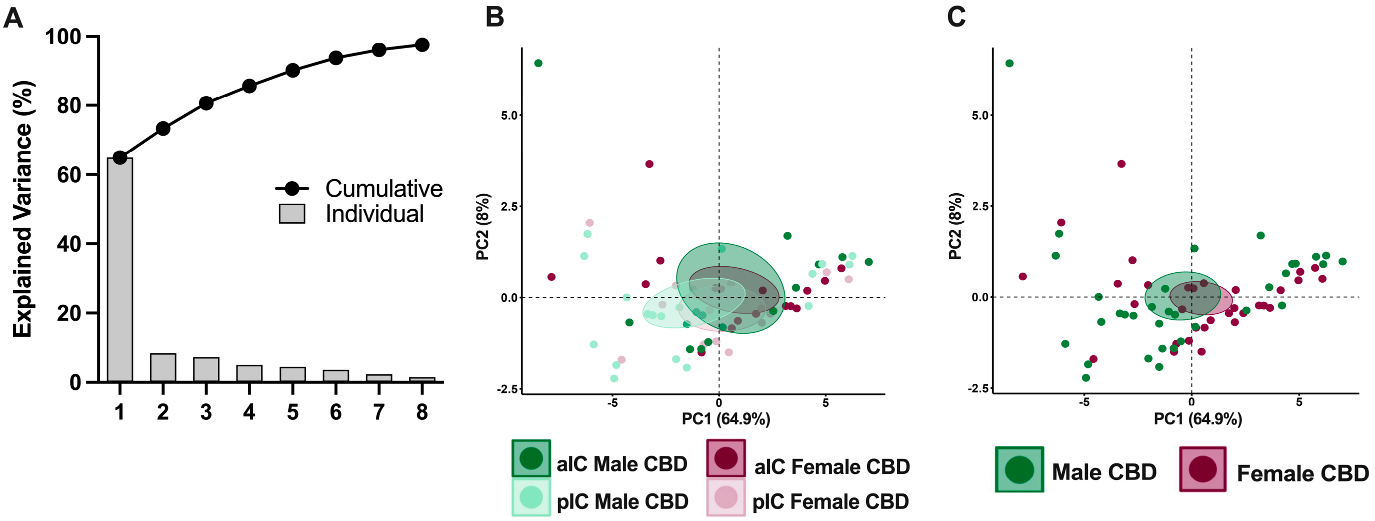

Supplemental Figure S3. Principal component analysis shows that gestational CBD abolished IC subregions differentiation in both sexes. Data were analyzed via PCA with membrane capacitance, rheobase, resting membrane potentials, neuronal excitabilities, and the voltage membrane's response to varying injected current steps as quantitative variables and cells as individuals. (A) Plotting the percentage of explained variance by each PC (histogram) reveals that most of the dataset's variance is explained by PC1 (64.9%) and PC2 (8%). The cumulative percentage of explained is represented by black dots. (B-C) Small dots represent individuals colored according to their belonging to one the following qualitative supplementary variables: sex and treatment(left) and sex (right). Each circle represents a cell plotted against its primary and secondary principal component (PC) scores. Ellipses represent the barycenter of individuals (i.e., mean) for each category, surrounded by its 95% confidence ellipses. (B) Group analysis revealed that this effect is driven by a loss of IC subregion differentiation following CBD in utero exposure. (C) PCA showed that in CBD males and females largely overlap. The sample size expressed as cells/Animal is: aIC CBD male = 15/8, pIC CBD male = 19/9, aIC CBD female = 17/7, pIC CBD female = 17/6.

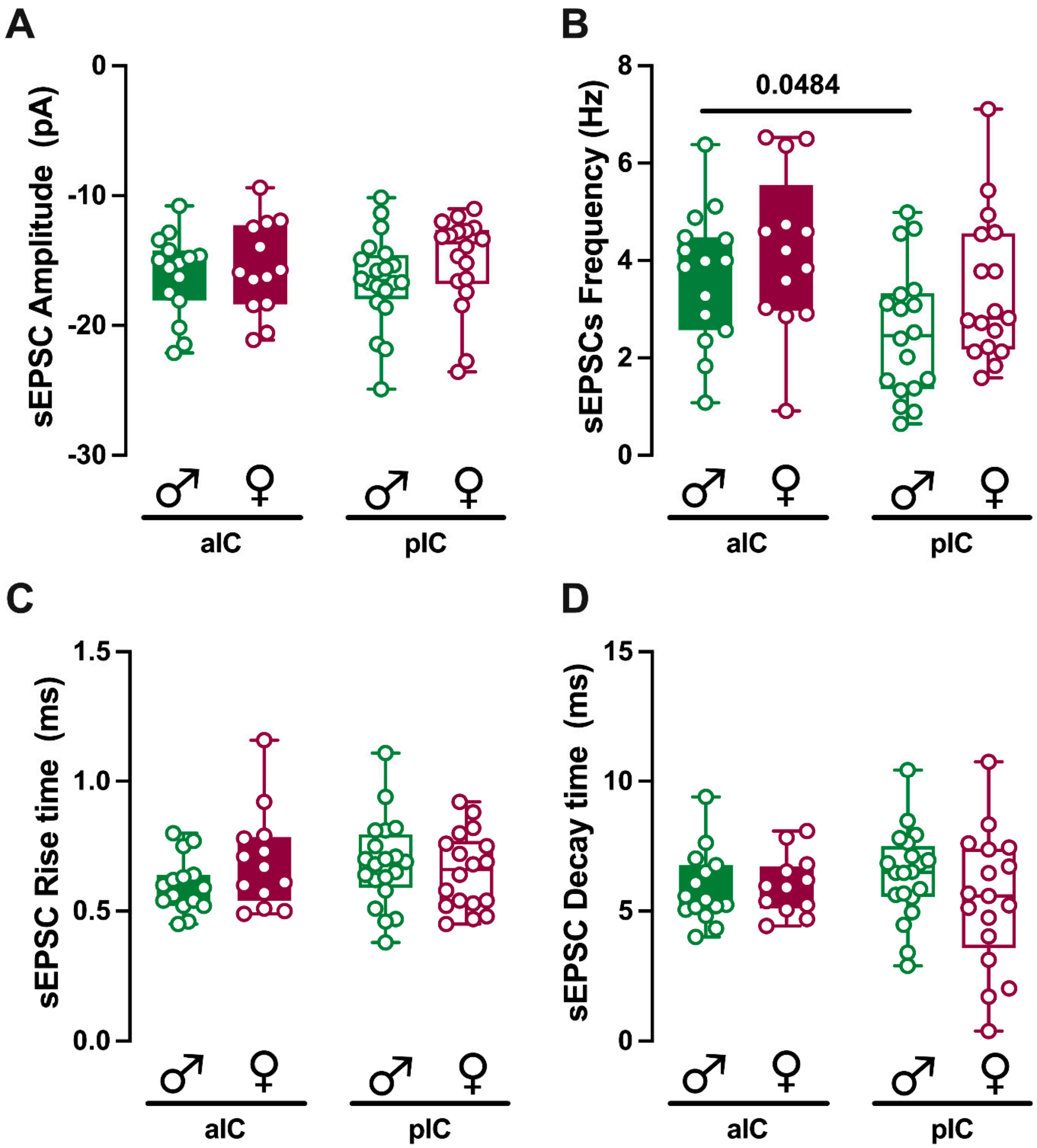

Supplemental Figure S4. Gestational CBD altered the excitatory transmission in a sex- and subregion-specific manner. (A) A quantitative analysis of mean amplitude and frequency in relation to area and sex demonstrated a similar amplitude of sEPSCs events. (B) In contrast, pIC excitatory events showed a lower frequency compared to aIC events in adult males only. (C-D) The kinetics of sEPSC were consistent across both areas and sexes. (A-D) Individual neurons are represented by single dots. Data are displayed as box and whisker plots (min., max., median). A two-way ANOVA followed by a Šídák multiple comparison test was used for data analysis. P-values <0.05 are indicated in the graphs. The sample size expressed as cells/Animal is: aIC male is represented as 9/14 in dark orange, pIC male as 16/27 in dark blue, aIC female as 6/13 in light orange, and pIC female as 12/17 in light blue.

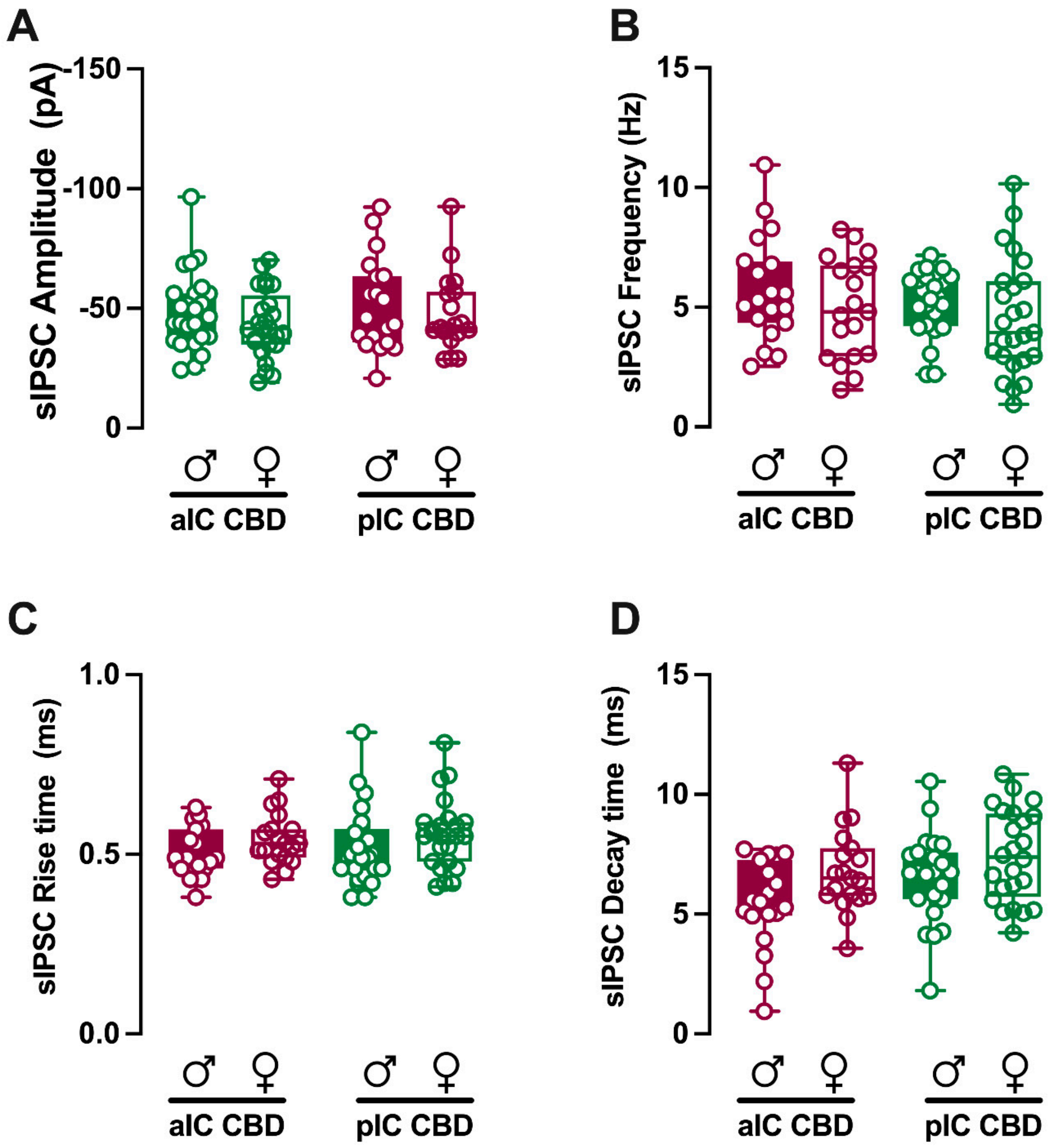

Supplemental Figure S5. The comparison of in utero Cannabidiol on the inhibitory transmission of CBD-exposed males and females. (A-D) No differences in the mean amplitude, frequency and the kinetics of sIPSCs in CBD progeny of both sexes were observed. (A-D) Data are displayed as box and whisker plots (min., max., median). (A-D) A two-way ANOVA followed by a Šídák multiple comparison test was used for data analysis. P-values <0.05 are indicated in the graphs. The sample size expressed as cells/Animal is: aIC CBD male = 25/13, pIC CBD male = 25/10, aIC CBD female = 19/13, pIC CBD female 19/10.
